# Understanding the laps and relapse process: in-depth interviews with individual who use methamphetamine

**DOI:** 10.1186/s13011-023-00548-9

**Published:** 2023-07-07

**Authors:** Faezeh Kaviyani, Mohammad Khorrami, Hamid Heydari, Malihe Namvar

**Affiliations:** 1 Addiction and Behavioral Sciences Research Center, North Khorasan University of Medical Sciences, Bojnurd, Iran; 2 Department of Counseling, Faculty of Education and Psychology, University of Isfahan, Isfahan, Iran; 3 Master of Science in Psychology, Bojnurd, Iran

**Keywords:** Methamphetamine, Laps, Relapse, Narcotics Anonymous

## Abstract

**Objective:**

The high rate of treatment failure is a common problem in the treatment of methamphetamine use. Therefore, the aim of this research is to identify the most common causes of relapse in methamphetamine users.

**Method:**

This is a qualitative study and of content analysis type. Information was collected using purposeful sampling and through semi-structured interviews and focus group discussions. The statistical population consisted of all people with the methamphetamine-use disorder in 2022 who were in the abstinence phase and participated in the meetings of the Narcotics Anonymous (NA) Center of Bojnord. Theoretical sampling continued until data saturation. A total of 10 one-on-one interviews were conducted, each lasting between 45 to 80 min. Additionally, two focus group interviews were conducted with six members in each group, lasting between 95 to 110 min and data saturation was achieved through these interviews. Data analysis was done using the content analysis method (Sterling). Recoding and Holsti's method were used to measure reliability; validity was then calculated through content validity assessment.

**Findings:**

The results of the thematic analysis showed that laps and relapse factors were identified and categorized into 5 organizing themes, including negative emotional states, positive emotional states, negative physical states, interpersonal factors, and environmental factors, consisting of 39 basic themes.

**Result:**

Identifying the risk factors leading to laps and relapse in methamphetamine users and increasing the knowledge in this field can lay the groundwork for preventive therapeutic interventions in this community.

## Introduction

Psychotropic drugs, including methamphetamine, are the most commonly used illegal drugs worldwide [1]. The number of users has increased from 24 to 34.2 million people between 2006 and 2017, making methamphetamine use a major concern for the World Health Organization (WHO) [2]. Therefore, the use of methamphetamine is one of the current concerns of the WHO (World Health Organization) [3]. A systematic study and meta-analysis revealed the 12-month prevalence of methamphetamine use in Iran to be 2.4% [4]. A study on 353 methamphetamine users found that mental problems, injuries, skin infections, and dental and oral hygiene problems affected approximately 18.7%, 18.4%, 11.11%, and 6.6% of users respectively [5]. Methamphetamine use has also been linked to heart failure and stroke [6, 7], and its long-term use can cause dysfunction and significant costs for families and communities [8, 9].

Diagnosing methamphetamine dependence, its symptoms, and treatment is more complicated than the diagnostic criteria in DSM IV-TR [10]. The high rate of relapse is a significant obstacle to the long-term effectiveness of substance use treatment programs [11].

Returning to drug use occurs in two forms; (a) complete return or relapse, and (b) partial return or lapse (slip). Lapse is defined as a temporally restricted and isolated pattern of drug use, whereas relapse is defined as a more severe and prolonged pattern of drug use [12].

Despite the implementation of advanced programs for treating substance use disorder, the recovery process, especially in cases of methamphetamine use, has been associated with high relapse rates [13]. Therefore, identifying factors related to lapses and relapses is crucial in providing effective treatments and managing them. A recent study by Huang et al. (2032) identified high craving as a risk factor for methamphetamine relapse [14]. Also, the study of Valencia and Peters (2023) showed that homesickness, lack of meeting and family support, fear of treatment, financial stress, stigma and social criticism, and boredom, are six barriers to recovery from methamphetamine use [15].

However, current treatment models such as Marlatt's cognitive-behavioral model of relapse [16], Marlatt's dynamic model of relapse [17], and reinstatement model [18] are based on studies conducted in the general population with substance use disorders. Therefore, further investigation is needed to develop specific treatment models for methamphetamine use disorder. It could be argued that these models were developed based on non-native studies, and since substance use is a human phenomenon influenced by culture, the cultural differences of various societies regarding lapses and relapse factors in treatment can impact the results. In Iran, the problem of drug use has specific local characteristics [19–21], Additionally, concerns exist regarding the rising prevalence of deaths caused by methamphetamine in Iranian society and the high incidence of substance use-related deaths in North Khorasan province, located in the northeast of Iran [22, 23]. Therefore, identifying factors associated with lapses and relapse among methamphetamine users is crucial. The present study aims to qualitatively investigate the lived experiences of methamphetamine users regarding lapses and relapse.

## Methods and data

This study utilized a qualitative approach through content analysis. The target population consisted of individuals with methamphetamine use disorder who were in the abstinence phase and attended Narcotics Anonymous Association meetings in Bojnord between 2022–2023. Purposeful snowball sampling was used until data saturation was reached. Inclusion criteria included a history of relapse within the past six months, age between 20–50 years, and no serious mental or medical illness. Methamphetamine use by mouth or injection was also required. Exclusion criteria included lack of consent and inability to express experiences. Interviews lasted between 45–80 min and interviewees' opinions were only mentioned through coding to comply with ethical considerations and spiritual principles of NA. Participants were informed that participation was optional, written consent-to-participate statements were obtained, and the purpose of audio recording was explained. If participants refused audio recording, notes were taken instead.

The primary method of data collection utilized in this study was semi-structured in-depth interviews and note-taking. A total of 22 individuals participated in the research, including 10 individual interviews and 2 focus group interviews with 6 members each. The interviews continued until data saturation was achieved, and the codes from previous interviews were both confirmed and repeated.

The interviews were conducted in addiction treatment centers and a quiet room without the presence of any third person. A special guide for semi-structured interviews and focus group discussions was used to obtain information on physical, psychological, occupational, academic, family, social, and other factors related to methamphetamine use.

The interview questions were compiled using main and general questions from the study as well as a review of related texts. Participants were asked about their experiences, feelings, and perceptions of lapses or returning to methamphetamine use. Additional information was obtained through STAR questions (situation, task, action, result) and w & h questions (who, what, where, when, why, how).

The interviewer was a psychologist specializing in substance use who held a briefing session for research participants to build trust before conducting the interviews. After each interview, the written transcript was reread several times by the interviewer.

### Data analysis

Analysis and note-taking were done concurrently with coding and continuous comparison. The process of conducting interviews continued until new codes were no longer obtained and the data reached saturation, at which point previous findings were confirmed through repetition. If new codes were obtained during an interview, another interview was conducted in a theoretical manner to ensure comprehensive sampling. Focus group interviews were also conducted and analysed using the same method. The Astrid-Sterling model [24], which has 3 stages and 6 steps, was used for data analysis. In addition, member checking, peer review, and recoding methods were used to measure the reliability of coding. As such, the text of the interviews and its summary were sent to a qualitative research method expert, and the problems related to the coding flow were resolved. To ensure the accuracy of the written account of the participants' comments and perspectives, the coding was returned to the participants and they were asked to confirm whether their experiences could be found in them. Based on the feedback of the participants, the coding was then modified and completed. Through re-coding and the Holsti method, coding reliability equal to 0.91 was obtained. The validity of the content analysis results was determined through content validity; the research results were sent to 11 experts in the field of substance use and qualitative research in the form of a 3-option (i.e. appropriate, somewhat appropriate, inappropriate) questionnaire. The Lavache coefficient of the questionnaire determined by the 11 experts was equal to 0.59 [25], the coefficients obtained for each of the themes were higher than this number, and the validity of the findings of the thematic analysis was confirmed. MAXQDA software was used to classify the contents.

From the total of 22 participants, 10 people participated in individual interviews and 12 formed two focused focus groups of 6 members each.

## Results

The demographic information of the investigated sample is reported separately for individual and focus group interviews in Table 1.

**Table 1:** History of methamphetamine use

	**One-on-ne interview(*N* = 10)**	**Focus Group 1 (*N* = 6)**	**Focus Group 2 (*N* = 6)**	**Total (*N* = 22)**
Addict’s Aged Main(SD)	28.33(± 3.33)	29.69(± 5.32)	30.12(± 6.41.)	29.44(± 5.11)
Age of Substance-use Onset Main(SD)	19.19(± 5.32)	18.88(± 4.29)	18.17(± 3.41)	17.92(± 4.04)
Substance-use Period (years) Main(SD)	6.38(± 3.49)	8.90(± 6.64)	7.83(± 5.65)	8.68(± 4.51)
No. of Relapses till now Main(SD)	3.16(± 1.31)	5.66(± 3.24)	5.26(± 2.43)	4.41(± 2.52)
No. of Lapss till now Main(SD)	4.89(± 6.6)	4.22(± 6.16)	4.05(± 4.83)	6.71(± 5.22)
Quitting times Main(SD)	5.86(± 2.37)	6.48(± 3.16)	5.77(± 4.87)	6.56(± 2.51)

The findings obtained from the content analysis of qualitative data revealed that a total of 5 organizing themes (evocation of positive emotions experienced during the use, negative emotional states. negative physical states, interpersonal factors, and environmental factors) and 39 sub-themes for the causes of laps and relapse of methamphetamine addicts have been identified and categorized, all of which are reported in Table 2.

**Table 2:** Basic themes found in relation to methamphetamine users' experience of relapse and relapse

Basic themes	Organizing themes
**Evocation of positive emotions experienced during the use**	Desire
	Positive emotions evoked by Positive fantasizing about use
	Positive emotions evoked by time and place of use
	Positive emotions evoked when witnessing friends use
	Cheerfulness & sense of freedom
	Self-expression
**Negative emotional states**	Regrets
	Remorse
	sadness
	Lack of Peace
	Intense fears
	loneliness
	Anger
	Depression
	Shame
	Anxiety
	disappointment
**Interpersonal factors**	Conversing with users
	Conflicts with spouse and children
	Being Chastised
	Being belittled by others
	Difficulty establishing intimate relationships
	Incompatibility of the individual with the guide
	Blending in with the crowd
	Dependence on substance using friends
	Being rejected
**Environmental factors**	Discriminatory treatment in the family
	Being forced to quit by parents
	Excessive parental control
	Bad friends
	Unemployment
	Occupation type
	Stigmatization
**Negative Physical States**	Heart palpitations and high blood pressure
	Nausea and vomiting
	Muscle pain and camps
	Fatigue
	Sleeplessness
	Illness & Injury

### Evocation of positive emotions experienced during the use

As can be seen in Table 2, the organizing theme of " *evocation of positive emotions experienced during the use* " and the 6 extracted basic themes related thereto are among the factors of Laps and Relapse. Regarding the basic theme of "desire", interviewe number 3 stated: "After some time had passed since quitting, I felt a compulsion and pressure from within to use again, and I came to the idea that I could start using potions without falling back into addiction again. I thought that with the skills I learned in NA's 12-step recovery program, I was ready to use ice every now and then without becoming dependent."

Regarding " evocation of positive emotions experienced during the use " interviewee number 8 said: " I reminiscence about the good times back when I used drugs. The good old days when I would use drugs with my friends around flash through my mind. Most of my friends are still smoking ice. The thought and longing for those times haunt and bother me. I said I'll take drugs and cry less and feel good again."

### Negative emotional states

Moreover, this study showed that 11 basic themes were identified for the organizing theme of " negative emotional states". For example, the 19-year-old interviewee with two laps experiences in relation to " disappointment " said: "I have had successive failures in my life and I have no hopes for the future. I have many economic and family problems that I am sure will not be solved in the future. In fact, the situation is getting worse than this. Imagining a vague and unsuccessful future caused me to use again." A 24-year-old participant mentioned "anxiety" as the cause of laps: " I was getting severe anxiety, I couldn’t help my worry and anxiety, and anyone who became friends with me would run away from me because I was very anxious and afraid of intimacy."

### Interpersonal factors

The next organizing theme was " interpersonal factors". The participants considered interpersonal factors among the most important and fundamental factors of laps. Finally, 9 themes were identified for interpersonal factors, an influential one of which was excessive dependence on substance using friends; the participants said that socializing with users peers is one reason for slipping and using methamphetamine again. One of the participants said: "My close friends don't appreciate me anymore because I stopped using methamphetamine, and sometimes they fight with me, and even some of my friends removed me from their group. Being alone is very hard and unbearable for me. Now, I don't even have anyone to grieve with."

### Environmental factors

Seven basic themes were identified for the organizing theme of " environmental factors". This organizing theme designates the factors that exist in the surrounding environment, society, and culture which constitute a high-risk factor instigating relapse. Some of the notions that people emphasized were frustrating situations, failure at work, and conflicts with the spouse and children. Unemployment was also one of the environmental factors occasionally mentioned by some participants. In this regard, one participant said: "The cause of most of our problems and misfortunes is unemployment. I have been using methamphetamine for two years, if I had a job and an income and I was busy, I would quit in less than a week." On the other hand, there were some who believed the type of job was one reason for relapse. For instance, heavy vehicle driving was often cited by participants as a reason for relapse. One of the participants commented: "I couldn't help myself, because anyone who drives a heavy vehicle on long and tiring roads will go back to smoking ice, because every time we were on the move, we had to spend at least two days on the road, so we would just lose control and use again."

### Negative physical states

The organizing theme of " negative physical states" and problems related to it was among the other reasons that the participants mentioned for returning to use. This organizing theme was comprised of 6 basic themes. Some were of the opinion that withdrawal from methamphetamine caused physical problems and pains, to reduce and eliminate which they would resort to using again. For example, one of the participants said: "After quitting methamphetamine, I experience numbness, chronic pain in the leg, blood circulation problems in the arms, and severe pain in the chest, and we end up having to use small amounts of drugs." The next participant considered lethargy and fatigue as the cause of slipping and stated: "After 50 days of quitting, I felt weak, lethargic, and extremely fatigued and I didn't feel like exercising or even walking, and at the same time I caught a cold and couldn't go to work, and if this had gone on, I would have lost my job."

## Discussion

The aim of this study was to explore the causes of lapses and relapses among people who use methamphetamine in Iran, using a qualitative approach. The results revealed that one of the major risk factors for returning to methamphetamine use is the “evocation of positive emotions experienced during the use ". This refers to situations where individuals seek to create or enhance positive emotions through methamphetamine use. Hendianti & Uthis (2018) Hendianti and Uthis (2018) found a correlation between positive emotional states and the risk of relapse [26]. The basic themes of positive emotional states include temptation to use, time and place of use, positive fantasizing, being cheerful, self-expression among friends, and lapses among friends. Marlatt (1996) suggests that substance use is often motivated by a desire to increase feelings of happiness, freedom, and pleasure or to experience the energizing effects of the drug. Therefore, individuals with methamphetamine use disorder may be more likely to relapse in response to stressors that trigger these desires. Another reason for lapses and relapses is cue reactivity, where individuals have a conditioned response to stimuli associated with drug use that draws them back into using again [27].

Another organizing theme identified in this research is " negative emotional states". This finding is consistent with previous studies [26, 28, 29]. that have shown that negative emotions and ineffective interpersonal situations are responsible for more than half of lapses and relapses, according to Marlatt's analysis (1996) [16]. Sinha (2007) also suggests that stress and related factors contribute to relapse [30]. Typically, three factors trigger relapse: negative moods, cognitive factors, and environmental factors [31]. Emotional relapse is often the first stage of relapse and occurs before the person realizes they may be at risk. During this stage, the individual experiences negative emotional states such as stress, anger, and anxiety. This can disrupt their diet and sleep cycle, reducing their desire to stay in treatment and recovery due to a lack of physical support systems [32]. These early emotional and physical warning signs are often dealt with by individuals with methamphetamine use disorder through drug use as a coping strategy, leading to relapse.

Another organizing theme identified in this research is " interpersonal factors". Witkiewitz and Marlatt have emphasized the role of interpersonal factors in relapse based on Marlatt's model [17]. Menon & Kandasamy (2018) suggest that positive social support can effectively prevent relapse and interpersonal conflicts, social pressure, and negative family behaviours [12]. The literature review also indicates that interpersonal stress and rejection sensitivity increase the risk of relapse in individuals with alcohol or drug use [33]. Mousali et al. (2021) found that family conflicts and having close friends and relatives who use substances are reasons for slipping into substance use among Iranian individuals who use substances [34]. Another study observed that individuals who did not return to substance use had better and closer relationships with their families and spouses [35]. Negative environments, such as lack of peace, being chastised, ridiculed, belittled, exposed to physical violence by relatives, and stigmatization, can damage an individual's recovery by damaging their sense of worth. This damage can cause the individual not to feel positive enough about themselves to protect themselves from engaging in harmful behaviours such as substance use. Resuming past relationships involved in substance use can also be a serious trigger leading to substance use.

The present study's results indicate that " environmental factors" are one of the most critical factors in returning to methamphetamine use. Family problems such as intense parental control, being forced by the family to quit, low family income, and discriminatory treatment in the family are some environmental factors influencing the return to substance use. Many studies have also found that bad family conditions and families with substance dependence are causes of returning to substance dependence [36, 37]. In their 2021 study, John and Kim found that having user friends can lead to drug use [38]. A meta-analysis conducted in Iran also revealed that environmental factors have a larger effect size on relapse (0.64) compared to individual factors (0.41) [39]. The role of environmental factors in relapse can be broken down into four dimensions. Firstly, individuals who use substances may turn to substances to cope with unpleasant emotions caused by environmental and social conflicts. Secondly, mistreatment, violence, and stigmatization can damage an individual's sense of self-worth, leading to harmful behaviours such as substance use. Thirdly, extreme family control and interference can push individuals towards drug use. Lastly, these factors prevent individuals from receiving positive social support which is crucial in preventing relapse [40, 41].

Another factor affecting relapse is negative physical states related to treatment. This includes heart palpitations, sweating, high blood pressure, nausea and vomiting, muscle pain and cramps, fatigue, sleeplessness, high irritability, illness and injury. Physical illness causes pain and stress which may lead individuals to turn to drug use as a means of reducing discomfort. In some studies, it has been found that individuals who use substances may turn to drugs again to alleviate the pain caused by chronic and incurable diseases or unexpected incidents [42, 43].

### Limitation

However, one limitation of this study is that some participants may not remember all their laps experiences, leading to the possibility of neglecting some relapses and their causes. Additionally, due to the principle of anonymity in the Addicts Anonymous association, some participants may refuse to disclose the truth. To address this limitation, participants were assured that their data and information would remain confidential and anonymous. However, another limitation is that the statistical population of this study was limited to the Narcotics Anonymous association in Bojnord, making it difficult to generalize these findings to other cultures and geographical conditions. Therefore, it is recommended to investigate the causes of laps and relapse in other substance users as well.

Based on the results obtained from this study, therapists and specialists should focus on factors such as negative emotional states, negative physical states, interpersonal factors, environmental factors, and positive emotional states in order to prevent laps and relapse and increase the duration of methamphetamine treatment.

Furthermore, as negative emotional states are identified as the primary underlying factors (12 categories) contributing to lapses and relapses in methamphetamine dependence, it is recommended that research be conducted to develop appropriate therapeutic interventions aimed at reducing these negative emotional states in affected individuals. Additionally, it is suggested that an educational package be created and evaluated for individuals surrounding those undergoing the quitting process, focusing on social and environmental factors as well as interpersonal conflicts, with the aim of reducing lapses and relapses. The authors of this study extend their gratitude to all the guides and participants who contributed to the interviews and data collection.

## Conclusion

Based on the findings of our study, it is clear that methamphetamine use is a complex issue that involves a range of factors, including emotional states, interpersonal relationships, environmental factors, and physical symptoms.

Overall, our study highlights the need for a comprehensive approach to addressing methamphetamine use that takes into account the multiple factors involved and the findings suggest that interventions aimed at preventing laps and relapse should address these underlying factors. For example, interventions could focus on helping individuals develop coping strategies for negative emotional states or improving their social support networks to reduce dependence on substance-using friends. Additionally, addressing environmental factors such as discrimination or excessive parental control may also be important in preventing laps and relapse. By taking a holistic approach to treatment, we can improve outcomes for individuals struggling with methamphetamine use and reduce the overall impact of this issue on society.

## Availability of data and materials.

## Data Availability

Data sets that were generated during the current study are available upon request from the corresponding author.

## References

[1] Abdoli N, Farnia V, Salemi S, Tatari F, Juibari TA, Alikhani M (2019). Efficacy of the Marlatt cognitive-behavioral model on decreasing relapse and craving in women with methamphetamine dependence: a clinical trial. J Subst Use.

[2] Drugs UNOo, Crime (2014). Global synthetic drugs assessment: amphetamine-type stimulants and new psychoactive substances.

[3] Degenhardt L, Singleton J, Calabria B, McLaren J, Kerr T, Mehta S (2011). Mortality among cocaine users: a systematic review of cohort studies. Drug Alcohol Depend.

[4] Abedi Gheshlaghi L, Sharifi H, Darabi M, Chegeni M, Khalili M, Noroozi A (2021). Prevalence of amphetamine-type stimulants use in Iran: a systematic review and meta-analysis. J Subst Use.

[5] Curtis EK (2006). Meth mouth: a review of methamphetamine abuse and its oral manifestations. Gen Dent.

[6] Zhao SX, Deluna A, Kelsey K, Wang C, Swaminathan A, Staniec A (2021). Socioeconomic burden of rising methamphetamine-associated heart failure hospitalizations in California from 2008 to 2018. Circ Cardiovasc Qual Outcomes.

[7] Westover AN, McBride S, Haley RW (2007). Stroke in young adults who abuse amphetamines or cocaine: a population-based study of hospitalized patients. Arch Gen Psychiatry.

[8] Minassian A, Henry BL, Iudicello JE, Morgan EE, Letendre SL, Heaton RK (2017). Everyday functional ability in HIV and methamphetamine dependence. Drug Alcohol Depend.

[9] Cumming C, Kinner SA, McKetin R, Li I, Preen DJD (2020). review a Methamphetamine use, health and criminal justice system outcomes: a systematic review. Drug Alcohol Rev.

[10] Liu L, Chui WH, Chai X (2018). A qualitative study of methamphetamine initiation among Chinese male users: Patterns and policy implications. Int J Drug Policy.

[11] Brown AR (2018). A systematic review of psychosocial interventions in treatment of opioid addiction. J Soc Work Pract Addict.

[12] Menon J, Kandasamy A (2018). Relapse prevention. Indian J Psychiatry.

[13] Nicolas C, Hofford RS, Dugast E, Lardeux V, Belujon P, Solinas M (2022). Prevention of relapse to methamphetamine self-administration by environmental enrichment: involvement of glucocorticoid receptors. Psychopharmacology (Berl).

[14] Huang M-C, Fang S-C, Lin C, Lin T, Chang H-M, Yang T-W, et al. Risk factors for relapse among methamphetamine users receiving a joint legal–medical treatment program as a diversion intervention: A one-year follow-up study. J Subst Use Addict Treat 2023:208955. 10.1016/j.josat.2023.208955 36804075

[15] Valencia MLC, Peters B. Factors related to motivation and barriers influencing treatment and recovery process of methamphetamine use disorder through in-depth, semi-structured, qualitative interviews. J Subst Use. 2023:1–7.

[16] Marlatt GA (1996). Models of relapse and relapse prevention.

[17] Witkiewitz K, Marlatt GA (2004). Relapse prevention for alcohol and drug problems: that was Zen, this is Tao. Am Psychol.

[18] Shaham Y, Shalev U, Lu L, De Wit H, Stewart JJP (2003). El modelo de reincorporación de la recaída de drogas: historia, metodología y hallazgos principales. Psicofarmacología.

[19] Hojjat SK, Kaviyani F, Akbari H, Norozi Khalili M, PROBLEMS RC. Sleep quality in heroin-dependent patients undergoing three types of maintenance therapy: The benefits promised by opium tincture maintenance therapy. Heroin Addict Relat Clin Probl 2020;22(5).

[20] Ghouchani HT, Lashkardoost H, Saadati H, Hojjat SK, Kaviyani F, Razaghi K (2021). Developing and validating a measurement tool to self-report perceived barriers in substance use treatment: the substance use treatment barriers questionnaire (SUTBQ). Subst Abuse Treat Prev Policy.

[21] Khorrami M, Pordelan N, Vakili S, Taghian F. Prediction of Coronavirus anxiety based on attachment styles, resilience, and life expectancy in drug users. Modern Care J 2022;19(1).

[22] Paknahad S, Akhgari M, Ghadipasha M (2021). Medicine, Pathology. An alarming rise in the prevalence of deaths with methamphetamine involved in Tehran, Iran 2011–2018. Forensic Sci Med Pathol.

[23] Hojjat SJ, Golmakanie E (2013). Incidence of death due to substance abuse documented in North Khorasan forensic medicine organization in 2008–2013. J N Khorasan Univ Med Sci.

[24] Attride-Stirling J (2001). Thematic networks: an analytic tool for qualitative research. Qual Res.

[25] Lawshe CH (1975). A quantitative approach to content validity. Pers Psychol.

[26] Hendianti GN, Uthis P (2018). Factors related to methamphetamine relapse risk among clients in the substance rehabilitation center of National Narcotics Board in West Java Indonesia. J Health Res.

[27] Zilberman N, Lavidor M, Yadid G, Rassovsky YJN (2019). Qualitative review and quantitative effect size meta-analyses in brain regions identified by cue-reactivity addiction studies. Neuropsychology.

[28] Rahman MM, Rahaman MM, Hamadani JD, Mustafa K, Shariful Islam SM (2016). Psycho-social factors associated with relapse to drug addiction in Bangladesh. J Subst Use.

[29] Brewer DD, Catalano RF, Haggerty K, Gainey RR, Fleming CBJA (1998). Research report a meta-analysis of predictors of continued drug use during and after treatment for opiate addiction. Addiction.

[30] Sinha R (2007). The role of stress in addiction relapse. Curr Psychiatry Rep.

[31] DiClemente CC (2018). Addiction and change: How addictions develop and addicted people recover.

[32] Anand D, Chen Y, Lindquist KA, Daughters SBJD, Dependence A (2017). Emotion differentiation predicts likelihood of initial lapse following substance use treatment. Drug Alcohol Depend.

[33] Leach D, Kranzler HR (2013). An interpersonal model of addiction relapse. Addict Disord Their Treat.

[34] Mousali AA, Bashirian S, Barati M, Mohammadi Y, Moeini B, Moradveisi L (2021). Factors affecting substance use relapse among Iranian addicts. J Educ Health Promot.

[35] Lavee Y, Altus D (2001). Family relationships as a predictor of post-treatment drug abuse relapse: a follow-up study of drug addicts and their spouses. Contemp Fam Ther.

[36] Zhang X, Zeng XJCP. Effects of family functioning on relapse among individuals with drug addiction in compulsory isolation: a chained mediation model. Curr Psychol 2021;1–11.

[37] Zeng X, Lu M, Chen M (2021). The relationship between family intimacy and relapse tendency among people who use drugs: a moderated mediation model. Subst Abuse Treat Prev Policy.

[38] Jun JH, Kim RW (2021). A study on the effects of cyber-bullying in adolescents on SNS addiction: focusing on the moderating effects of friendship. J Kor Acad-Industr Coop Soc.

[39] Safari S, Kamali A, Dehghani FS, Esfahani M (2014). Meta-analysis of comparing personal and environmental factors effective in addiction relapse (Iran, 2004–2012). Sci Quart Res Addict.

[40] Castañeda AM, Lee C-S, Kim Y-C, Lee D, Moon JY (2018). Addressing opioid-related chemical coping in long-term opioid therapy for chronic noncancer pain: a multicenter, observational, cross-sectional study. J Clin Med.

[41] Ganesh S, Kandasamy A, Sahayaraj US, Benegal V (2018). Behavioral activation and behavioral inhibition sensitivities in patients with substance use disorders: a study from India. Indian J Psychiatry.

[42] Khazaee-Pool M, Pashaei T, Nouri R, Taymoori P, Ponnet K (2019). Understanding the relapse process: exploring Iranian women’s substance use experiences. Subst Abuse Treat Prev Policy.

[43] Jakubczyk A, Ilgen M, Kopera M, Krasowska A, Klimkiewicz A, Bohnert A (2016). Reductions in physical pain predict lower risk of relapse following alcohol treatment. Drug Alcohol Depend.

